# Evaluation of Rural Primary Health Care in Western China: A Cross-Sectional Study

**DOI:** 10.3390/ijerph121113843

**Published:** 2015-10-29

**Authors:** Manli Wang, Haiqing Fang, Ghose Bishwajit, Yuanxi Xiang, Hang Fu, Zhanchun Feng

**Affiliations:** School of Medicine and Health Management, Tongji Medical College, Huazhong University of Science and Technology, Wuhan 430030, Hubei, China; E-Mails: wangmanli1237@hust.edu.cn (M.W.); fanghaiqing@hust.edu.cn (H.F.); Brammaputram@gmail.com (G.B.); stephenhsiang@gmail.com (Y.X.); tongjifh@163.com (H.F.)

**Keywords:** rural primary health care, western China, evaluation, differences

## Abstract

*Purpose:* China’s Ministry of Health has enacted Rural Primary Health Care Program (2001–2010) (HCP) guidelines to improve the quality of people’s health. However, the program’s success in Western China remains unevaluated. Thus, this study aims to begin to fill that gap by analyzing the provision and utilization of Rural Primary Health Care (RPHC) in Western China. *Methods:* A cross-sectional study was conducted to collect secondary data on the socio-economic characteristics, system construction, services use and implementation of RPHC, and the residents’ health status of the sampled areas. Four hundred counties from 31 provinces in China were selected via stratified random sampling, including 171 counties from 12 Western provinces. Twenty-seven analysis indicators, covering system construction, services use and implementation of RPHC were chosen to assess Western China’s primary health quality. Analysis of Variance (ANOVA) and Least Significant Difference (LSD) methods were used to measure the RPHC disparities between Western and Eastern and Central China. Technique for Order Preference by Similarity to Ideal Solution (TOPSIS) was used to rank Western, Eastern and Central internal provinces regarding quality of their RPHC. *Results:* Of the 27 indicators, 13 (48.15%) were below the standard in Western China. These focused on rural health service system construction, Chinese medicine services, and public health. In the comparison between Western, Central and Eastern China, 12 indicators had statistical significance (*p <* 0.05), and using LSD to compare between Western and Eastern China, all indicators were statistically significant (*p <* 0.05), demonstrating regional disparities. Xinjiang Province’s RPHC ranked highest overall, and Yunnan Province ranked the lowest, indicating the internal differences within the 12 Western provinces; and Western provinces’ C_i_ value was lower than that of Eastern and Central China overall. *Conclusion:* Western China’s RPHC has proceeded well, but remains weaker than that of Eastern and Central China. Differences within Western internal provinces threaten the successful implementation of RPHC.

## 1. Introduction

In the 1970s, a survey by the World Health Organization (WHO) found that, due to the unfair allocation of health resources, more than half of the World’s population did not have access to suitable health care services [1]. This demographic segment was mainly located in rural areas and urban slums. Faced with this situation, in 1977, the 30th World Health Congress proposed the goal of “everyone will enjoy health care by 2000” [2]. In 1978, the international health conference hosted by WHO and the United Nations International Children’s Emergency Fund (UNICEF) promulgated the Declaration of Alma-Ata [3], which put forward the concept of “primary health care (PHC)”, which became accepted as the basic strategy to realize the goal of universal attainment of an acceptable level of health by the year 2000. In 1981, the WHO developed a series of assessment indicators (Table 1) to help each member state examine and evaluate their PHC programs [4]. Rural Primary Health Care (RPHC) is an important measure for the Chinese government to apply PHC to rural areas [5], and it is conceptualized to provide services to rural residents, and thus to improve health, prevent disease, heal injuries and provide rehabilitation services for rural residents, with the aim of ensuring their access to adequate health care.

RPHC is the most basic health care services that rural residents can access, and is a key indicator of social equality [6,7]. RPHC should be flexible enough to adapt to rural economic and social development, and every resident should have access to it. RPHC may well be one of the most significant systemic and ideological health reforms of our time [8]. RPHC can be considered a philosophy; an approach to the delivery and development of services and first contact health service. It is based on a social (rather than bio-medical) model of health care, with accessibility and affordability of services as primary objectives. Countries with stronger RPHC systems have demonstrably more efficient, effective, and equitable health care [9].

PHC was the theme of the World Health Report in 2008, 30 years after the Alma-Ata Declaration, and has been the topic of a series of significant conferences around the world [10,11]. In response to WHO’s target of developing PHC, in 2002, the Chinese Ministry of Health (MOH) promulgated the Rural Primary Health Care Program (2001–2010) guidelines, which put forward the general goal of improving the level of health and quality of life for rural residents [12]. In 2009 the proposal to rebuild a good RPHC system as a central goal in Chinese health-care reform was announced [13]. After this, in order to promote RPHC, China took several measures including increasing financial investment in RPHC, strengthening rural health care professional teams, carrying out projects relating to health education and improving drinking water and sanitation across the country, reinforcing management of maternal and child health services, strengthening supervision of public health, and making medicine and related health products available [14]. After more than 10 years of effort, China’s RPHC has entered into a new development stage, as has rural residents’ health level and life quality [15].

**Table 1:** The evaluation indicators of PHC formulated by WHO.

Kinds	Indicators
Health policy indicators	(1) Political obligation for everyone’s health
	(2) Resources allocation
	(3) Equity degree of health resources allocation
	(4) Community participation for realizing the goal of everyone will enjoy health care
	(5) Organization system and management
Social-economic indicators related with health	(1) Population growth rate
	(2) Gross national product
	(3) Income distribution
	(4) Working conditions (the possibility of obtaining a job)
	(5) Adult literacy rate
	(6) Housing
	(7) Food supply
Health care provision indicators	(1) Availability
	(2) Accessibility
	(3) Economic and cultural accessibility
	(4) Health service utilization
	(5) Popularization of primary health care
	(6) Popularization of referral system
	(7) Health manpower
Status of people’s health indicators	(1) Child nutritional status
	(2) Infant mortality
	(3) Child mortality
	(4) Maternal mortality
	(5) Expected life

Western China includes 12 provinces (or municipalities and autonomous regions directly under the Central Government), namely Chongqing, Sichuan, Guizhou, Yunnan, Guangxi, Shaanxi, Gansu, Qinghai, Ningxia, Tibet, Xinjiang, and Inner Mongolia. With a vast geographical area and a sparse population, Western China contains nearly 42,000,000 rural residents and is populated by Chinese minority ethnic groups. Western China is an underdeveloped area, whose area output value median *per capita* and *per capita* net income of the agricultural population were much lower than those of Eastern and Central China. There are significant differences in both the natural and social environment between Western China and the Eastern and Central China, and likewise in the application of the RPHC. Additionally, both at home and abroad, research on RPHC in Western China is insufficient. Therefore, it is necessary to evaluate RPHC work in Western China. This research therefore aims to analyze both the achievements and deficiencies of RPHC work in Western China, confirm the factors that affected the RPHC implementation, then put forward targeted policy recommendations for improvements.

## 2. Methods

### 2.1. Sample Survey and Data Collection

A cross-sectional survey was conducted in 2010 using stratified random sampling. First, each of the 31 provinces in China were divided into three layers- the upper, middle and lower layer- according to the social and economic development level, and from each layer 20% of counties were randomly extracted. This gave a total of 400 counties sampled, including 79 eastern counties, 150 central counties and 171 western counties. Second, secondary data relating to the implementation status of RPHC, such as the socio-economic characteristics, system construction, services use and implementation of RPHC, and the residents’ health status of the sampled areas were collected, and personal in-depth interviews were conducted in the Health Bureau and Centers of Diseases Control (CDC) of these 400 counties.

### 2.2. The Selection of Indicators

Based on the indicators developed by WHO (Table 1), through three rounds of expert consultation (Delphi) and pre-investigations, the Chinese government finally determined 27 indicators and their standard values, and the Kendall’s coefficient of concordance W of this indicator system was 0.447, which had undergone a test of statistical significance (*p <* 0.01) (W is the Kendall’s coefficient of concordance of different experts’ marks on the indicators). These 27 assessment indicators covered three aspects: system construction, services use and implemented results of RPHC. Together, the indicators covered 10 domains, namely government support, rural health service system construction, basic medical management specification, basic public health services, health surveillance, maternal and child health, environmental health, health education, the new rural cooperative medical care, and the health status of residents [16].

### 2.3. Data Analysis

First, the basic natural and social situation of these 400 counties in China was described. Following this, the secondary data concerning the 27 assessment indicators of Rural Primary Health Care (RPHC) were analyzed using SPSS 13.0 to determine the unadjusted overall mean, which were compared with the standard values of RPHC in China. Third, ANOVA and LSD methods were used to measure the RPHC difference between Western China and Eastern and Central China. Finally, the traditional comprehensive TOPSIS evaluation method was used to rank RPHC work among internal provinces in three areas of China. TOPSIS is a comprehensive analysis method, which makes a ranking through a positive ideal solution and a negative ideal solution. The result that is both closest to the positive ideal solution and farthest away from the negative ideal solution is regarded as the optimal solution [17]. TOPSIS is a multi-index evaluation method, which can avoid the unicity of a single-index evaluation. At the same time, compared with other multi-index evaluation methods, TOPSIS can calculate simply, rank clearly, has no limitation on data types, sample size and index numbers, and it is not affected by the subjective influence of researchers.

### 2.4. TOPSIS Process

Taking the comprehensive evaluation of Western China’s PRHC work as an example, the TOPSIS process has six activities, which are listed below:

(1) $$F=\left[\begin{array}{cccc} f_{11} & f_{12} & ... & f_{1m}\\ f_{21} & f_{22} & ... & f_{2m}\\ ... & ... & ... & ...\\ f_{n1} & f_{n2} & ... & f_{nm} \end{array}\right]$$

Activity 1: Establish a decision matrix for the ranking. The structure of the matrix can be expressed as above, where m=27, and n=12.

In this study, an initial matrix composed of RPHC evaluation indicators of the 12 provinces in Western China was built.

Activity 2: Calculate the normalized decision matrix Z (= $\left[Z_{\text{ij}}\right]$). The normalized value $\left[Z_{\text{ij}}\right]$ is calculated as:

(2) $$Z_{ij}=\frac{{\text{f}}_{ij}}{\sqrt{\sum_{j=1}^{\begin{array}{l} n \end{array}}f_{ij}^{2}}}$$

where j=1...n; i=1...m.

Activity 3: Calculate the weighted normalized decision matrix by multiplying the normalized decision matrix by its associated weights. The weighted normalized value $V_{ij}$ is calculated as:

(3) $$V_{ij}=W_{ij}Z_{ij}$$

where $W_{j}$ represents the weight of the j^th^ attribute or criterion. In this case, all the indicators have the same $W_{ij}$, so:

(4) $$V_{ij}=Z_{ij}$$

Activity 4: Determine the positive ideal solution and the negative ideal solution:

(5) $$Z^{+}=(\max Z_{i1},\max Z_{i2}...\max Z_{im})$$

(6) $$Z^{-}=(\min Z_{i1},\min Z_{i2}...\min Z_{im})$$

where the positive ideal solution $Z^{+}$ is composed of the maximum value of each column in Z, and the negative ideal solution $Z^{-}$ is composed of the minimum value of each column in Z. In this case, the positive ideal solution presents that each indicator of every western province in China has the best value; the positive ideal solution presents that China’s western provinces’ each indicator has the lowest value.

Activity 5: Calculate the separation measures, using the dimensional Euclidean distance:

(7) $$\begin{array}{l} D_{i}^{+}=\sqrt{\sum_{i=1}^{m}{\left(\max Z_{ij}-Z_{ij}\right)}^{2}}\;\\ D_{i}^{-}=\sqrt{\sum_{i=1}^{m}{\left(\min Z_{ij}-Z_{ij}\right)}^{2}}\; \end{array}$$

In this study, $D_{i}^{+}$ is the Euclidean distance from the indicator group of every Western province to the positive ideal solution, and $D_{i}^{-}$ is the Euclidean distance from the indicator group of every Western province to the negative ideal solution.

Activity 6: calculate the relative closeness to the optimal solution and rank the alternatives in descending order. $C_{i}$ is the relative closeness of the evaluation object to the optimal solution, and it is also the basis of ranking. The value of $C_{i}$ is from 0 to 1. The larger $C_{i}$’s value, the better the status of the RPHC in western provinces of China:

(8) $$C_{i}=\frac{D_{i}^{-}}{D_{i}^{+}+D_{i}^{-}}$$

## 3. Results

### 3.1. Basic Social-Economic Overview of the Sample Counties in Eastern, Central and Western China

The sample area consists of 400 counties from 31 provinces in China. The total population of Western China is 52,360,000, which is less than that of Eastern (52,570,000) and Central China (74,970,000). The terrain of Western China is also more complicated than that of other two areas. Among the 171 sample counties of Western China, mountainous areas account for 46.78%, while 18.13% of the counties comprise hills, and 16.96% plateaus. In terms of land area included in the study, the coverage of Western China was 12 times than that of Eastern China, and five times than that of Central China. In these 171 sample counties, rural residents’ median net income *per capita* was 3585 RMB, which is lower than the national average of 4850 RMB，the Eastern China of 7050 RMB, and the Central China of 4496 RMB (Table 2).

### 3.2. Overview of RPHC Work Reaching the Standard in Eastern, Central and Western China

To better assess China’s RPHC, this article adopted the index reference values shown in Table 3, according to the HCP guidelines.

**Table 2:** Characteristics of the 400 sample counties in Eastern, Central and Western China in 2010.

Items	Kinds	Eastern China	Central China	Western China	Total
Sample areas	Provinces	11	8	12	31
	Sample counties (*n*)	79	150	171	400
Population	Total population	52570	74970	52360	179900
	The rural population (thousand)	40690	61260	42000	143950
	The non-rural population (thousand)	11880	13710	10360	35950
Terrain	Plain (%)	41.77	37.33	13.45	28.00
	Hill (%)	24.05	28.00	18.13	23.00
	Mountainous area (%)	30.38	32.67	46.78	38.25
	Plateau (%)	0.00	0.00	16.96	16.96
	Others (%)	3.80	2.00	4.68	8.19
Cover area	Cover area（km^2^）	151389	355814	1813816	2321019
Economy	Median area output value *per capita* (RMB)	23860	13122	10252	13274
	*Per capita* net income of the agricultural population (RMB)	7050	4496	3585	4578

**Table 3:** Indicators for 400 sample counties in Eastern, Central and Western China’s RPHC compared with reference values (%).

RPHC	Eastern China (*n* = 79)		Central China (*n* = 150)		Western China (*n* = 171)	
	Reference	Mean	Reference	Mean	Reference	Mean
**Construction of rural primary health care service system indicators**						
1. Designating the RPHC as a government work target and socioeconomic development plan	100	94.20	100	97.70	100	98.10
2. The increased proportion of government’s investment to rural health	25	29.32 ^a^	25	23.90 ^a^	25	23.56
3. The proportion of township hospitals administrated by county	95	95.19 ^a^	95	99.73 ^a^	95	99.04 ^a^
4. The coverage rate of rural medical institutions	100	100.00 ^a^	90	100.00 ^a^	85	100.00 ^a^
5. The proportion of practicing physicians and practicing assistant physicians providing clinical services in the clinics	100	86.51	100	83.16	100	71.05
6. The proportion of practicing physicians and practicing assistant physicians providing clinical services in the township hospitals	85	13.80	75	8.22	60	9.94
**Use of rural primary health care services indicators**						
7. The proportion of township hospitals providing traditional Chinese medicine services	50	23.94	50	29.63	50	38.74
8. The proportion of village clinics providing traditional Chinese medicine services	50	23.65	50	30.52	50	30.15
**Use of rural primary health care services indicators**						
9. The rate of rural doctors using basic drugs directory	85	87.27 ^a^	85	84.01	85	82.17
10. The qualification rate of township hospitals using and managing disposable medical supplies	95	96.06 ^a^	90	90.64 ^a^	85	93.53 ^a^
11. The qualification rate of clinics using and managing disposable medical supplies	85	89.66 ^a^	85	80.08	85	84.09
12. The rate of major chronic disease management	50	47.05	35	39.07 ^a^	20	30.93 ^a^
13. The coverage rate of DOTS	95	98.10 ^a^	95	98.90 ^a^	95	99.31 ^a^
14. The rate of planned immunization	95	99.12 ^a^	90	98.04 ^a^	85	97.49 ^a^
15. The qualification rate of food hygiene	80	89.48 ^a^	80	90.95 ^a^	80	78.49
16. The qualification rate of public place health	90	88.27	90	100.00 ^a^	90	85.61
17. The qualification rate of occupational health	95	100.00 ^a^	95	86.13	95	98.30 ^a^
18. The maternal hospital delivery rate	80	99.43 ^a^	60	89.04 ^a^	50	75.23 ^a^
19. The care coverage rate of children under the age of 7	80	87.49 ^a^	70	70.35 ^a^	60	72.88 ^a^
20. The access rate to tap water	75	84.39 ^a^	60	56.75	50	49.39
21. The access rate to sanitary latrines	65	67.18 ^a^	55	59.89 ^a^	35	42.97 ^a^
22. The basic health knowledge awareness rate	80	83.02 ^a^	70	79.53 ^a^	60	65.50 ^a^
23. The participation rate of the new rural cooperative medical system (NCMS)	75	80.21 ^a^	75	80.09 ^a^	75	79.56 ^a^
24. The utilization rate of funds from NCMS	60	86.34 ^a^	60	64.88 ^a^	60	61.56 ^a^
**Results of the implementation of rural primary health care indicators**						
25. The decrease in the infant mortality rate	20	4.07	20	7.70	20	9.64
26. The decrease in the maternal mortality rate	25	6.39	25	19.30	25	46.84 ^a^
27. The decreased proportion of children under age 5 mortality rate	20	4.50	20	8.12	20	11.41

Note: **^a^** Indicators reaching the reference standard.

The indicators reaching or exceeding the reference values were regarded as being up to standard. Of these 27 indicators, Eastern, Central and Western China respectively had 17 (62.96%), 15 (55.56%) and 14 (51.85%) indicators classifiable as “up to standard”. These three areas’ common indicators “up to standard” were: the coverage rate of rural medical institutions, the rate of planned immunization, the basic health knowledge awareness rate, the utilization rate of funds from NCMS, and others. In addition, the Eastern and Central China reached standard too concerning the increased proportion of government’s investment to rural health, the qualification rate of food hygiene and so on. This showed that Western China’s RPHC has progressed well [16], but the rate of being up to standard is still lower than that of Eastern and Central China. Moreover, 48.15% of indicators in Western China were found to be “not up to standard”, which will restrict the ability of the RPHC system to provide basic health services. These indicators were mainly focused on the rural health service system construction, Chinese medicine services, Children’s access to health care, and other public place health problems (Table 3).

### 3.3. RPHC’s Disparities among Western, Central and Eastern China

This paper analyzed the RPHC’s disparities with ANOVA method. From Table 4, we can see that 12 indicators were statistically significant in their variance (*p <* 0.05), which were the proportion of practicing physicians and practicing assistant physicians providing clinical services in the township hospitals, the proportion of township hospitals providing traditional Chinese medicine services, the coverage rate of Diphtheria, Pertussis, Tetanus vaccine (DPT), the maternal hospital delivery rate and so on. However, the paired comparison using LSD method to compare Central and Western Chinese RPHC only had two indicators with statistical significance, namely the proportion of township hospitals providing traditional Chinese medicine services and the maternal hospital delivery rate, whilst all these 12 indicators were statistically significant (*p <* 0.05) in the comparison between Western and Eastern China.

**Table 4:** Differences in RPHC among Western, Central and Eastern China based on ANOVA and LSD.

RPHC	ANOVA		LSD	
			*p*	
	F	*p*	East (*n* = 79) & West (*n* = 171)	Central (*n* = 150) & West (*n* = 171)
**Construction of rural primary health care service system**				
The coverage rate of rural medical institutions	0.491	0.617	0.554	0.643
The proportion of practicing physicians and practicing assistant physicians providing clinical services in the township hospitals	5.372	0.011 *	0.030 **	0.056
The proportion of practicing physicians and practicing assistant physicians providing clinical services in the clinics	1.661	0.208	0.141	0.776
**Use of rural primary health care services**				
The proportion of township hospitals providing traditional Chinese medicine services	5.180	0.012 *	0.005 **	0.043 **
The proportion of village clinics providing traditional Chinese medicine services	2.002	0.154	0.057	0.531
The rate of major chronic disease management				
Hypertension system management rate	0.341	0.714	0.416	0.734
Diabetes system management rate	0.170	0.845	0.566	0.838
The rate of planned immunization				
the coverage rate of Bacillus Calmette-Guerin (BCG)	1.362	0.273	0.131	0.889
the coverage rate of DPT	5.003	0.014 *	0.005 **	0.572
the coverage rate of Measles vaccine	6.015	0.007 *	0.002 **	0.299
the coverage rate of Polio vaccine	4.702	0.170	0.005 **	0.360
the coverage rate of Hepatitis B vaccine	8.298	0.001 *	0.000 **	0.334
The qualification rate of food hygiene	1.929	0.164	0.093	0.132
The qualification rate of public place health	0.635	0.537	0.319	0.983
The qualification rate of occupational health	1.599	0.220	0.111	0.198
The maternal hospital delivery rate	20.168	0.000 *	0.000 **	0.002 **
The care coverage rate of children under the age of 7	6.317	0.005 *	0.002 **	0.547
The access rate to tap water	13.253	0.000 *	0.000 **	0.310
The access rate to sanitary latrines	10.296	0.000 *	0.000 **	0.091
The basic health knowledge awareness rate	1.860	0.174	0.136	0.100
**Results of the implementation of rural primary health care**				
The decrease in the infant mortality rate	4.740	0.017 *	0.006 **	0.551
The decrease in the maternal mortality rate	6.916	0.004 *	0.001 **	0.051
The decreased proportion of children under age 5 mortality rate	4.026	0.029 *	0.009 **	0.184

Notes: * Statistically significant among three groups; ** Statistically significant between two groups.

As for all the indicators with statistical significance, the indicator value of Eastern and Central China is higher than that of Western China. Thus, it can be seen that China’s RPHC presents significant regional differences, especially between Eastern and Western China.

### 3.4. Ranking of Internal Provinces’ RPHC Work in Eastern, Central and Western China

According to the six activities of TOPSIS process in the method, the ranking of internal provinces’ RPHC work in Eastern, Central and Western China was calculated. Taking the TOPSIS analysis of Western China’s RPHC for example, the results of the process are below:

(1) The initial matrix of RPHC of 12 Western provinces had been established (Table 5).
(2) The standardized matrix has been established (Table 6).
(3) Because the weight of all indicators is the same, the weighted matrix is equal to the standardized matrix.
(4) The maximum value of each column in Table 6 was taken out to constitute the positive ideal solution:
  Z^+^ = (0.28, 0.39, 0.43, 0.28, 0.34, 0.26, 0.54, 0.41, 0.32, 0.29, 0.32, 0.62, 0.30, 0.29, 0.31, 0.31, 0.28, 0.36, 0.36, 0.38, 0.43, 0.34, 0.32, 0.34, 0.68, 0.65, 0.65)
  And the minimum value of each column in Table 6 was taken to constitute the negative ideal solution:
  Z^-^ = (0.28, 0.11, 0.24, 0.26, 0.10, 0.00, 0.09, 0.05, 0.23, 0.24, 0.24, 0.10, 0.28, 0.27, 0.25, 0.24, 0.24, 0.13, 0.18, 0.09, 0.17, 0.01, 0.23, 0.19, 0.01, 0.06, 0.04)
(5) D^+^ was calculated, which is the Euclidean distance from every province’s indicator group to Z^+^, so as D^-^, which is the Euclidean distance from every province’s indicator group to Z^-^;
(6) Ci was calculated, which is the relative closeness to the optimal solution and the basis of comprehensive ranking of 12 Western provinces’ RPHC work (Table 7).

.

**Table 5:** The initial matrix of RPHC in 12 Western provinces of China.

Province	The Indicators of RPHC in China																										
	1	2	3	4	5	6	7	8	9	10	11	12	13	14	15	16	17	18	19	20	21	22	23	24	25	26	27
Tibet	1.00	0.35	0.94	1.00	0.28	0.00	0.36	0.35	0.98	1.00	0.92	0.25	1.00	0.99	0.81	0.73	1.00	0.33	0.54	0.53	0.77	0.63	0.91	0.67	0.21	0.22	0.26
Inner Mongolia	1.00	0.27	1.66	0.96	0.87	0.15	0.37	0.33	0.72	0.96	0.83	0.50	1.00	0.98	0.90	0.89	1.00	0.90	0.85	0.33	0.37	0.73	0.77	0.58	0.05	0.10	0.60
Xinjiang	1.00	0.36	1.05	0.95	0.37	0.19	0.16	0.06	0.87	0.96	0.93	0.16	1.00	0.95	0.86	0.85	1.00	0.85	0.77	0.71	0.40	0.72	0.82	0.56	0.09	0.25	0.04
Guangxi	1.00	0.31	1.06	1.00	0.84	0.09	1.00	0.30	0.87	1.00	0.82	0.51	1.00	1.00	1.00	0.85	0.90	0.50	0.45	0.20	0.44	0.02	0.80	0.60	0.83	0.07	0.06
Ningxia	1.00	0.21	1.00	1.00	0.86	0.10	1.00	0.50	0.92	0.95	0.87	0.25	1.00	1.00	1.00	0.96	0.83	0.45	0.51	0.16	0.73	0.09	0.70	0.75	0.01	0.08	0.47
Yunnan	1.00	0.10	0.98	1.00	0.79	0.01	0.45	0.35	0.76	0.93	0.84	0.15	1.00	0.98	0.89	0.90	1.00	0.66	0.70	0.62	0.45	0.71	0.71	0.64	0.11	0.32	0.13
Gansu	1.00	0.18	1.01	1.00	0.63	0.09	0.34	0.39	0.85	0.96	0.87	0.15	1.00	0.98	0.92	0.86	1.00	0.73	0.67	0.47	0.41	0.68	0.85	0.47	0.13	0.31	0.12
Guizhou	1.00	0.21	0.99	1.00	0.78	0.06	0.27	0.17	0.83	0.97	0.88	0.72	1.00	0.97	0.88	0.86	1.00	0.57	0.50	0.68	0.36	0.78	0.66	0.79	0.79	0.72	0.08
Qinghai	1.00	0.30	1.13	1.00	0.69	0.08	0.48	0.44	0.90	0.97	0.95	0.17	1.00	0.98	0.94	0.86	1.00	0.74	0.75	0.63	0.48	0.79	0.89	0.78	0.21	0.21	0.26
Shaanxi	1.00	0.23	1.08	1.00	0.63	0.15	0.28	0.26	0.85	0.83	0.76	0.19	1.00	0.99	0.88	0.82	1.00	0.89	0.86	0.39	0.30	0.76	0.82	0.74	0.09	0.18	0.12
Sichuan	1.00	0.20	1.00	1.00	0.80	0.11	0.34	0.35	0.73	0.85	0.77	0.17	1.00	0.95	0.86	0.83	1.00	0.77	0.73	0.52	0.42	0.74	0.64	0.50	0.08	0.36	0.15
Chongqing	1.00	0.22	1.01	1.00	0.92	0.11	0.36	0.32	0.94	1.00	1.00	0.92	1.00	0.99	0.95	0.84	1.00	0.92	0.89	0.69	0.68	0.81	0.75	0.82	0.10	0.34	0.12

Note: 1, 2, 3… 27 are the indicators of RPHC in China.

**Table 6:** The standardized matrix of RPHC in 12 Western provinces of China.

Provinces	The Indicators of RPHC																										
	1	2	3	4	5	6	7	8	9	10	11	12	13	14	15	16	17	18	19	20	21	22	23	24	25	26	27
Tibet	0.28 **^a^**	0.38	0.24 **^b^**	0.28	0.10 **^b^**	0.00 **^b^**	0.20	0.29	0.32 **^a^**	0.29 **^a^**	0.29	0.17	0.28 **^b^**	0.28	0.25 **^b^**	0.24 **^b^**	0.28 **^a^**	0.13 **^b^**	0.21	0.28	0.43 **^a^**	0.26	0.32 **^a^**	0.28	0.17	0.20	0.28
Inner Mongolia	0.28 **^b^**	0.29	0.43 **^a^**	0.27	0.32	0.22	0.20	0.27	0.23 **^b^**	0.28	0.27	0.34	0.28	0.28	0.28	0.29	0.28	0.36	0.34	0.17	0.21	0.30	0.27	0.24	0.04	0.09	0.65 **^a^**
Xinjiang	0.28	0.39 **^a^**	0.27	0.26 **^b^**	0.14	0.26	0.09 **^b^**	0.05 **^b^**	0.28	0.28	0.30	0.11	0.28	0.27	0.26	0.28	0.28	0.34	0.31	0.38	0.22	0.30	0.29	0.23	0.07	0.23	0.04 **^b^**
Guangxi	0.28	0.33	0.27	0.28 **^a^**	0.31	0.13	0.54 **^a^**	0.24	0.28	0.29	0.26	0.35	0.28	0.29 **^a^**	0.31	0.28	0.25	0.20	0.18 **^b^**	0.11	0.24	0.01 **^b^**	0.28	0.25	0.68 **^a^**	0.06 **^b^**	0.06
Ningxia	0.28	0.22	0.26	0.28	0.32	0.15	0.54	0.41 **^a^**	0.30	0.28	0.28	0.17	0.28	0.29	0.31 **^a^**	0.31 **^a^**	0.24 **^b^**	0.18	0.20	0.09 **^b^**	0.41	0.04	0.25	0.31	0.01 **^b^**	0.07	0.51
Yunnan	0.28	0.11 **^b^**	0.25	0.28	0.29	0.02	0.24	0.29	0.25	0.27	0.27	0.10 **^b^**	0.28	0.28	0.27	0.29	0.28	0.26	0.28	0.33	0.25	0.30	0.25	0.27	0.09	0.29	0.14
Gansu	0.28	0.20	0.26	0.28	0.23	0.13	0.18	0.32	0.28	0.28	0.28	0.10	0.30 **^a^**	0.28	0.28	0.28	0.28	0.29	0.27	0.25	0.23	0.28	0.30	0.19 **^b^**	0.11	0.28	0.13
Guizhou	0.28	0.22	0.26	0.28	0.29	0.09	0.14	0.14	0.27	0.29	0.28	0.48	0.28	0.28	0.27	0.28	0.28	0.22	0.20	0.36 **^a^**	0.20	0.32	0.23 **^b^**	0.33 **^a^**	0.64	0.65 **^a^**	0.09
Qinghai	0.28	0.31	0.29	0.28	0.25	0.11	0.26	0.36	0.29	0.29	0.30	0.11	0.28	0.28	0.29	0.28	0.28	0.29	0.30	0.33	0.27	0.33 **^a^**	0.32	0.33	0.17	0.19	0.28
Shaanxi	0.28	0.25	0.28	0.28	0.23	0.22 **^a^**	0.15	0.22	0.28	0.24 **^b^**	0.24 **^b^**	0.13	0.28	0.28	0.27	0.26	0.28	0.35	0.34	0.21	0.17 **^b^**	0.32	0.29	0.31	0.07	0.16	0.12
Sichuan	0.28	0.21	0.26	0.28	0.29	0.16	0.18	0.29	0.24	0.25	0.25	0.11	0.28	0.27 **^b^**	0.26	0.27	0.28	0.31	0.29	0.27	0.24	0.31	0.23	0.21	0.07	0.32	0.17
Chongqing	0.28	0.23	0.26	0.28	0.34 **^a^**	0.15	0.19	0.26	0.31	0.29	0.32 **^a^**	0.62 **^a^**	0.28	0.28	0.29	0.27	0.28	0.36 **^a^**	0.36 **^a^**	0.36	0.38	0.34	0.27	0.34	0.08	0.31	0.13

Notes: **^a^** presents the maximum of each column，and it’s one of the elements of the positive ideal solution; **^b^** presents the maximum of each column, and it’s one of the elements of the negative ideal solution.

**Table 7:** Ranking of RPHC work in Eastern, Central and Western provinces of China.

Provinces	D^+^	D^-^	C_i_	Ranking
Eastern China				
Shanghai	0.60	1.49	0.71	1
Zhenjiang	0.94	1.07	0.53	2
Beijing	1.23	1.33	0.52	3
Tianjin	1.20	1.23	0.51	4
Fujian	1.12	1.13	0.50	5
Shandong	1.09	1.02	0.48	6
Heilongjiang	1.13	1.03	0.48	6
Jilin	1.22	1.00	0.45	8
Jiangsu	1.19	0.89	0.43	9
Liaoning	1.20	0.82	0.40	10
Guangdong	1.29	0.69	0.35	11
Hebei	1.31	0.66	0.34	12
Hainan	1.55	0.61	0.28	13
Central China				
Anhui	0.46	1.31	0.74	1
Shanxi	1.05	0.95	0.47	2
Henan	1.13	0.77	0.40	3
Hunan	1.10	0.74	0.40	3
Hubei	1.06	0.64	0.38	5
Jiangxi	1.26	0.41	0.24	6
Western China				
Xinjiang	1.24	1.26	0.50	1
Inner Mongolia	1.33	1.00	0.43	2
Guangxi	1.37	0.99	0.42	3
Guizhou	1.36	0.94	0.41	4
Chongqing	1.37	0.94	0.41	4
Ningxia	1.43	0.91	0.39	6
Qinghai	1.38	0.78	0.36	7
Tibet	1.48	0.68	0.32	8
Shaanxi	1.51	0.68	0.31	9
Sichuan	1.49	0.65	0.30	10
Gansu	1.51	0.60	0.28	11
Yunnan	1.55	0.59	0.27	12

Notes: **D^+^** is the distance from the indicator group of every Western province to the positive ideal solution; **D^−^** is the distance from the indicator group of every Western province to the negative ideal solution; **C_i_** is the relative closeness to the optimal solution and is also the basis of ranking.

In Table 5, the row represents the value of the 27 RPHC indicators for each Western province, and the column represents the same one indicator’s value for the 12 western provinces. The initial matrix is the raw data, and is the basis of the establishment of the standard matrix

As for Table 6, the row represents the standardized value of the 27 RPHC index for each Western province, and the column represents the same one indicator’s standardized value for the 12 Western provinces. The standardized matrix is the basis of calculating the positive ideal solution and the negative ideal solution, so is the calculation of the Euclidean distance.

As it could be seen from Table 7, as for the ranking of RPHC work, in Eastern China, the top three provinces are Shanghai, Zhejiang and Beijing, and Hainan and Hebei province rank the worst in Central China, Anhui Province is the best, and Jiangxi Province is the worst; among the 12 Western provinces in China, Xinjiang Province’s PHC work ranked the highest overall, followed by the Inner Mongolia Autonomous Region and the Guangxi Zhuang Autonomous Region. The three lowest ranked provinces were Yunnan, Gansu and Sichuan. This suggests that large differences in RPHC existed within Eastern, Central and Western China, and in these three regions, the higher the provinces’ social-economic level, the higher the ranking of their RPHC work. We can also find that the C_i_ value of provinces within Eastern and Central China is relatively higher as a whole, whose range is 0.4–0.75, and the internal provinces’ C_i_ value of Western China is relatively lower as a whole, whose range is 0.20–0.50. This indicates that the RPHC work within China’s Eastern and Central provinces is relatively better than that of the internal provinces in Western China.

## 4. Discussion

Since the HCP guidelines were implemented, China has adopted a range of strategies to promote RPHC work, with the active cooperation of provincial and municipal governments as well as Chinese residents. This study also found that RPHC in Western China has proceeded relatively well, which demonstrates the effectiveness of China’s RPHC policy. However, despite the good aspects, this study has found that Western China’s RPHC still faces many challenges to overcome, for example, continuing to improve the rate of “up to the standard” of RPHC, reducing the regional gap of RPHC work between Western and Eastern and Central China, and promoting the common development of Western internal 12 provinces.

In order to improve the rate of “being up to standard” of RPHC of Western China, first of all, the Chinese government should strengthen the construction of rural health services system. The rural health service system is the basis of RPHC [18,19], and health-care personnel are the key to rural health service system construction. Western China’s rural health service system construction is weak, especially regarding the severe shortage of medical practitioners or physicians’ assistants. This is because Western China is poor, and RPHC personnel in Western China are not paid well, thus few personnel are willing to undertake rural, grass-roots work. Another reason postulated is that health authorities and primary health institutions have no real autonomy, resulting in difficulties in improving the quality of primary health teams. Fixing these would not only require that China increase the government’s investment in western regions’ rural health, but also require putting the RPHC into the government work target and socioeconomic development plan, improving the treatment of RPHC personnel, and giving health officers freedom to manage health-care personnel and health services system construction.

Secondly, the Chinese government should develop the traditional Chinese medicine (TCM) in Western China. A low proportion of TCM application in rural health institutions is another challenge. Western China is a region populated by ethnic minorities, which are a key resource in maintaining TCM knowledge and diversity. However, Western medicine tends to replace TCM very quickly [20,21]. TCM is claimed to be more conducive to curing the foundations of some diseases, and has been associated with fewer adverse effects according to Geng [22] and Jia [23]. According to Yao [24] and Qian [25], the government should thus enact policies to support TCM; however, there is limited evidence to support this. In addition, we found that the use of disposable supplies in rural western regions was low. This was mainly due to the fact that rural doctors’ use of disposable medical supplies was not standardized. Therefore, China should focus more on the standardized management of disposable medical supplies whilst training rural doctors.

Moreover, the level of children health care in Western China should be improved. Because of the low population density, the access and availability of basic public health services and basic medical services’ availability to children was low in Western China. Furthermore, the Western region’s economy is relatively poor, and thus financial barriers sometimes prevented children from accessing what medical care was available. Similarly, as a result of the harsh natural and socio-economic environment, water penetration in western rural areas was also low. All of these public problems need the Chinese government improve emphasis on Western China’s RPHC work, and special support is also necessary for improving the access of Western rural residents to RPHC.

To narrow the regional gap between Western and Eastern and Central RPHC is also a huge challenge. The result that RPHC work in Western China is weaker than that in the Eastern and Central China, especially weaker than that of Eastern China is similar to the research of Guo *et al.* and Al *et al.* [26,27]. As for the reasons, according to a review of work by Chinese scholars, the health service supply system in poor Western China lacks sufficient operating funds and service capabilities, and most rural families have a low educational level, leading to poor access to necessary knowledge and health information [28]. At the same time, Western China also faces a shortage of health workers, and a poorly formulated health system human resource structure, where the medical staff’s overall level of education and job title are far below that of the national average level [29].

Therefore, in order to promote coordinated development of national rural primary health care and reduce regional differences, China should institute a range of new measures. First, increase support efforts and ensure adequate sanitation and financial investment in rural primary health care work in Western China. Second, strengthen training and assessment of health-care personnel in the county, township and village. This will contribute to improving the overall quality and accessibility of rural primary health care services in Western China. Third, strengthen supervision and management of RPHC work, and improve the medical staff’s awareness of service and work quality were deemed necessary. Finally, it is also important to strengthen the education and publicity work of RPHC, enhance rural residents’ health-care awareness, and improve their life quality and health level [30].

Narrowing the gap of Western China’s internal provincial RPHC is another huge challenge. From the ranking results of Western China’s internal provincial RPHC, that Xinjiang and Mongolia’s RPHC was much better than other provinces’ is mainly because these governments attach great importance to it, and invest more in health-care work. Other areas of Western China should follow the example of Xinjiang and Mongolia, and adopt a policy of continuous improvement of their RPHC. At the same time, all the Western provinces in China would benefit from enhanced cooperation, communication and exchange, with a view to common development. From the comparison of ranking results of Western China and Eastern and Central China’s internal provincial RPHC, on one hand, we think that Western China should learn the experience of Eastern and Central China in implementation of RPHC, to improve the level of RPHC from the whole; on the other hand, the Chinese government should encourage the Eastern and Central provinces to concert with the Western poor provinces in the process of implementing RPHC program, to narrow down the RPHC differences internal provinces.

We think the regional and internal provincial differences of RPHC in China were affected not only by the natural environment and social economy and provincial government’s support, but also by health resources allocation essentially. The unfairness of health resources allocation played the most important role in this. Survey results by Li *et al.* corroborate this [31]. Liu *et al*. also believed that the main reason for unfair health resources allocation was the regional internal differences, which far outweighed the differences between regions [32]. As a consequence, the Chinese government should not only attach great importance to the overall RPHC work in western regions, but also promote common development of all the provinces. The Chinese government should establish a policy that covers the provision of funds and equipment to the provinces whose environments are harsh, and whose economic development lags behind. In addition, it is necessary to encourage provinces where RPHC work has developed well to better support poorer provinces and regions with less successful implementation. Provision of the most basic health care services to everyone in Western China will only be possible through the common development of all the Western provinces’ RPHC.

## 5. Limitations

There are several limitations of this study. First, with a cross-sectional study, the survey results only indicate the implementation status of RPHC in Western China in 2010, which may have left the progress of RPHC in Western China in past 10 years and recent 5 years unstudied. Second, this study evaluates the implementation status of RPHC, which may have left the influence factors of RPHC not being explored. Third, we study the implementation results of RPHC from the point of view of the RPHC supplier, thus making the specific use and individual differences of users of RPHC unstudied. Finally, this study compares the implementation status of RPHC in the Eastern, Central and Western China, but doesn’t make a comparative analysis between China and other developed or developing countries concerning the implementation results of RPHC. These limitations are what we should continue to explore in the future.

## 6. Conclusions

RPHC is critical for improving the quality of rural people’s health in rural China. In this study, we intended to evaluate the implementation status of RPHC in Western China, and examine the disparity of RPHC in Eastern, Central and Western China and the difference within internal 12 Western provinces. The study revealed that Western China’s RPHC work has progressed well as a result of the last 10 years of effort, but is still weaker than that of Eastern and Central China. Differences within Western internal provinces undermine the implementation of primary health care. It is, therefore, important to promote the implementation of key and complex projects, and promote common development within western internal provinces would be a logical measure. Based on the findings, we suggest that the government further strengthen system construction of RPHC, improve its coverage and provide more primary health-care services to the rural population and narrow the RPHC gap of three regions and 12 internal Western provinces, to meet the complex healthcare needs of different rural residents.

## Abbreviations

ANOVA: Analysis of Variance
TOPSIS: Technique for Order Preference by Similarity to Ideal Solution
CDC: Centers of Diseases Control
HCP: Rural Primary Health Care Program (2001–2010) guidelines
LSD: Least Significant Difference
MOH: Chinese Ministry of Health
RPHC: Rural primary health care
BCG: Bacillus Calmette-Guerin
DPT: Diphtheria, Pertussis, Tetanus vaccine
TCM: Traditional Chinese medicine
UNICEF: United Nations International Children’s Emergency Fund
WHO: The World Health Organization
